# The Microbiota-Gut-Brain Axis in Health and Disease and Its Implications for Translational Research

**DOI:** 10.3389/fncel.2021.698172

**Published:** 2021-07-15

**Authors:** Melanie Anna Schächtle, Stephan Patrick Rosshart

**Affiliations:** Department of Medicine II (Gastroenterology, Hepatology, Endocrinology, and Infectious Diseases), Medical Center – University of Freiburg, Faculty of Medicine, University of Freiburg, Freiburg, Germany

**Keywords:** gut microbiota, gut-brain axis, germ-free rodent, short-chain fatty acids, wildling, microglia, Alzheimer’s disease, stroke

## Abstract

Over the past decades, microbiome research has evolved rapidly and became a hot topic in basic, preclinical and clinical research, for the pharmaceutical industry and for the general public. With the help of new high-throughput sequencing technologies tremendous progress has been made in the characterization of host-microbiota interactions identifying the microbiome as a major factor shaping mammalian physiology. This development also led to the discovery of the gut-brain axis as the crucial connection between gut microbiota and the nervous system. Consequently, a rapidly growing body of evidence emerged suggesting that the commensal gut microbiota plays a vital role in brain physiology. Moreover, it became evident that the communication along this microbiota-gut-brain axis is bidirectional and primarily mediated by biologically active microbial molecules and metabolites. Further, intestinal dysbiosis leading to changes in the bidirectional relationship between gut microbiota and the nervous system was linked to the pathogenesis of several psychiatric and neurological disorders. Here, we discuss the impact of the gut microbiota on the brain in health and disease, specifically as regards to neuronal homeostasis, development and normal aging as well as their role in neurological diseases of the highest socioeconomic burden such as Alzheimer’s disease and stroke. Subsequently, we utilize Alzheimer’s disease and stroke to examine the translational research value of current mouse models in the spotlight of microbiome research. Finally, we propose future strategies on how we could conduct translational microbiome research in the field of neuroscience that may lead to the identification of novel treatments for human diseases.

## Introduction

All mammals are multicellular organisms that are not only composed of their own individual cells but also of the microorganisms that inhabit all epithelial barrier sites and well-defined, distinct ecological niches such as the gastrointestinal tract, the skin, the respiratory tract, and the genitourinary system. In this context, it was shown that not just the number of microbial cells in the body (Sender et al., 2016) but also their genetic material vastly exceeds the corresponding human counterparts which emphasizes the potential of the microbiota to influence the host (Li et al., 2014; Xie et al., 2016; Lloyd-Price et al., 2017). Microbiota is defined as all microbes present within an ecological niche such as the gut, whereas the microbiome is the combination of the microbiota and their genes (Berg et al., 2020). By now we know that the vast majority of microorganisms inhabit the gastrointestinal tract (Berg, 1996). The gut microbiota is a complex and diverse ecosystem comprising microbes of all kingdoms such as bacteria, archaea, viruses, fungi, protozoa, and the meiofauna. It is acquired through vertical transmission and environmental exposure. Importantly, these microbes do not just passively colonize the gut of their hosts, in contrast, they established an intimate, mutually beneficial, symbiotic relationship over eons of co-evolution. The host provides its microbial subtenants with a habitat as well as nutrients, and in return, the microbes offer various health benefits (Dethlefsen et al., 2007; Ley et al., 2008; Sommer and Bäckhed, 2013). For instance, the commensal gut microbiota plays an essential role for the synthesis of several vitamins (e.g., vitamins K and B), it provides energy for the host in terms of short-chain fatty acids (SCFAs, e.g., butyrate) by fermentation of otherwise indigestible carbohydrates as well as fibers and it is involved in the metabolism of bile acids, sterols and the deactivation of xenobiotics (Cummings and Macfarlane, 1997; O’Hara and Shanahan, 2006). Taken together, the gut microbiome plays a vital role in virtually every aspect of mammalian physiology, specifically in the development, maturation, homeostasis, and ultimately the function of the immune system in health and disease (Cerf-Bensussan and Gaboriau-Routhiau, 2010; Hooper et al., 2012; Belkaid and Hand, 2014; Zheng et al., 2020). Hence, the mammalian phenotype is driven by a combination of the host genome and the microbial genome aka microbiome, together referred to as the metagenome, which is also acknowledged in the holobiont theory or the meta-organism concept (Norman et al., 2014; Stappenbeck and Virgin, 2016).

The source of this increasing understanding regarding host-microbiota interactions certainly was the development of high-throughput sequencing technologies, which enabled scientists to perform metagenomic studies (Arnold et al., 2016). Naturally, there was also growing evidence of a bidirectional communication between the central nervous system (CNS) and the gastrointestinal microbiota called the microbiota-gut-brain axis, which turned out to be an essential contributor to overall brain physiology. There are complex and manifold ways the microbiota uses to communicate and influence the host via this axis (Collins et al., 2012). Microbiota can have a direct impact on the production of metabolic precursors like tryptophan or the synthesis of neurotransmitters like serotonin and dopamine or they produce SCFAs (Diaz Heijtz et al., 2011; Sampson and Mazmanian, 2015; Yano et al., 2015). SCFAs can also act in an epigenetic fashion by inhibiting histone deacetylases (Wu et al., 2012). Further, the microbiota and the CNS are linked by the release of microbial-associated molecular patterns (MAMPs) (Sampson and Mazmanian, 2015). MAMPs include bacterial derived molecules like double-stranded RNA, lipopolysaccharide and lipoproteins which are recognized by different receptors, mainly belonging to Toll-like receptors (Akira and Hemmi, 2003). Additionally, the gut is connected to the CNS through the vagus nerve and enables a direct communication through neurochemicals (Forsythe et al., 2014). Jonathan Kipnis even proposed a defining role of the immune system to sense microorganisms and to deliver relevant information about them to the brain via different immune signaling molecules like cytokines (Kipnis, 2018). However, bio-active microbial molecules and metabolites appear to play a dominant role in mediating the communication along the gut-brain axis.

Animal models were crucial in highlighting and understanding the manifold impact of microbes on the nervous system in development, maturation, aging and homeostasis such as changes in expression of neurotrophic factors, NMDA receptor subunits in the hippocampus (Sudo et al., 2004; Bercik et al., 2011; Diaz Heijtz et al., 2011), impaired blood-brain barrier function, increased myelination in the prefrontal cortex (Braniste et al., 2014; Hoban et al., 2016) as well as learning and memory (Gareau et al., 2011; Neufeld et al., 2011; Clarke et al., 2013; Swann et al., 2020). Further, there is also some evidence for a role of microbiota in psychiatric disorders like depression and anxiety (Foster and McVey Neufeld, 2013), autism spectrum disorder (ASD) (Krajmalnik-Brown et al., 2015; Marrone and Coccurello, 2019), schizophrenia (Severance et al., 2016; Marrone and Coccurello, 2019) as well as neurological diseases such as Alzheimer’s disease (AD) (Kowalski and Mulak, 2019), Parkinson’s disease (PD) (Keshavarzian et al., 2015) and stroke (Durgan et al., 2019; Battaglini et al., 2020). Additionally, gut microbiota has been suggested to control the maturation and function of microglia, the CNS resident immune cells (Erny et al., 2015; Matcovitch-Natan et al., 2016; Louveau and Kipnis, 2018; Thion et al., 2018). Since microglia lie at the interface between environmental signals and the brain, they may be a critical link between the microbiome and the CNS. Actually, microglia has been shown to contribute to CNS diseases such as ASD, AD, multiple sclerosis (MS), and PD, all of which are also affected by gut microbiota (Sharon et al., 2016).

Thus, conventional laboratory mice, especially germ-free (GF), antibiotic-treated, gnotobiotic and specific-pathogen-free (SPF) mice were an invaluable tool for proof-of-principal studies that highlighted the profound impact of microbiota on mammalian physiology. Through mouse models, scientist learned about the importance of microbiota in health and disease, thereby identifying microbiota as a potential target for novel treatment strategies above and beyond the field of neuroscience. However, aside from these valuable proof-of-principal studies, there are only few success stories where basic biological principals discovered in mice could be directly translated into the human system, creating breakthroughs in translational medicine such as immune checkpoint inhibitors (Littman, 2015) as well as the discovery of MHC restriction (Zinkernagel and Doherty, 1974a,b). In general, laboratory mouse models appear to be fairly limited when it comes to predicting complex physiological responses of humans. Hence, the transition from preclinical studies in mice to bedside practice in humans rarely works (Mestas and Hughes, 2004; von Herrath and Nepom, 2005; Payne and Crooks, 2007; Seok et al., 2013; Shay et al., 2013; Hay et al., 2014; Mak et al., 2014), especially in neurosciences (Garner, 2014; Pound and Rebecca, 2020). There is a vast body of literature illustrating that the conventional laboratory mouse has not only misdirected clinical approaches consuming trillions of research funding (Wong et al., 2019), but also let to catastrophically failed human clinical trials (Fisher et al., 1996; Suntharalingam et al., 2006). The consequent and crucial question is how to improve the translational research value of animal models and increase the safety and success rate of transitioning treatments into the clinic?

Here, we want to highlight how the gut microbiota impacts the development, maturation, aging, homeostasis and function of the brain. Further, we show how microbiota potentiates disease states in AD and stroke in a negative as well as positive way. Subsequently, we exemplify the translational research value of the laboratory mouse in the context of AD and stroke research. Finally, we speculate on how the recent progress in the development of translational research models may be utilized in the field of translational microbiome neuroscience to maximize the clinical benefit and identification of novel treatments.

## The Influence of the Gut Microbiome on the Development of the Nervous System

**FIGURE 1:**
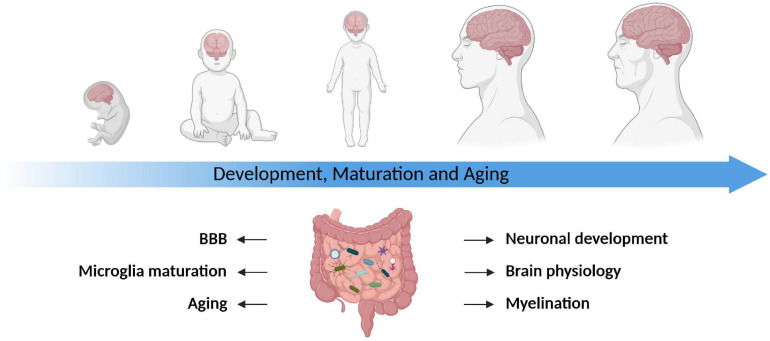
The gut microbiota modulates the development, maturation, and aging of the CNS. The gut microbiota plays a pivotal role throughout all stages of life. It impacts brain physiology, myelination, blood–brain barrier function (BBB) as well as microglia maturation and contributes to the process of aging. Table lists corresponding references, all studies are primarily based on rodent data.

The microbial colonization of all mammalian organisms starts at birth with the passage of the newborns through the birth canal and the exposure to the vaginal microbiome of their mothers. Subsequently, the first contact between the immune system and the microbiota happens at all epithelial barrier sites of the body, thereby shaping immune tolerance to commensal microbes and establishing barrier integrity. The infant’s microbiome develops and stabilizes within 2–3 years in humans (Stecher, 2015) and by the age of 2–3 weeks in mice (Schloss et al., 2012). Importantly, this early window of life is not only characterized by critical phases of development and growth involving barrier sites themselves, but also involves virtually all organs distal to barrier sites and multi-organ structures such as the immune system (Arrieta et al., 2014). Remarkably, the brain increases its size more than 50% during the first 3 months of life and reaches 90% of the size of an adult organ within the first 5 years (Tierney and Nelson, 2009). The vast majority of neuronal development happens during this pivotal phase (O’Mahony et al., 2017). Since the periods of microbiota development coincides with the majority of brain development, researchers hypothesized early on that these times of parallel development may be of high biological relevance. This was subsequently confirmed by various studies as illustrated in Figure 1. Today, the commensal microbiota is being acknowledged as an integral part of the gut-brain axis and recognized to be an important modulator of the brain’s physiology during all stages of life in health and disease. In addition, we know that the neuronal development is not only significantly impacted, but also orchestrated by the maternal microbiota (De Palma et al., 2015; Gomez de Agüero et al., 2016; Fleshner et al., 2017; Sarker and Peleg-Raibstein, 2018; Pasciuto et al., 2020; Vuong et al., 2020). The microbiota might act as an expected input to calibrate the development of the gut–brain axis and its absence or disruption during specific developmental windows is hypothesized to influence neuronal as well as neuro-cognitive development and ultimately behavior. Animal studies of social interactions provided strong evidence for microbial modulation of cognition and behavior. A robust relationship between social behavior and the microbiota was observed across species and led to the hypothesis that this relationship has an evolutionary basis (Cowan et al., 2020).

The influence of gut microbiota in neurodevelopment was recognized since the early 2000s by experiments using GF, antibiotic-treated, gnotobiotic and SPF mice. Researchers disrupted the microbiota of mice through antibiotic treatment which resulted in animals displaying several neurological problems (Sudo et al., 2004; Neufeld et al., 2011; Clarke et al., 2013; De Vadder et al., 2014; Daneman and Prat, 2015; Buffington et al., 2016) like impaired social behaviors (Cryan and Dinan, 2012; Buffington et al., 2016), reduced anxiety like behavior (Neufeld et al., 2011; Cryan and Dinan, 2012; Clarke et al., 2013; Cryan et al., 2019) and increased motor and rearing activity (Diaz Heijtz et al., 2011; Arentsen et al., 2015). The observed impairment of brain development and behavior could be ameliorated by reconstitution of newborn mice with a diverse and intact microbiome (Cryan and Dinan, 2012; O’Mahony et al., 2017; Cryan et al., 2019).

Swann and Heijtz have previously shown that the gut microbiota can influence brain physiology, for instance through the regulation of neurotransmission and synaptogenesis (Diaz Heijtz et al., 2011) as well as through the modulation of the metabolic profiles of the prefrontal cortex and hippocampus of rodents (Swann et al., 2017). In their newest study, they characterized the neurobiochemical profile of the forebrains of mice during three key postnatal developmental stages, co-occurring with the maturation of the gut microbiota. They could show that gut microbial-derived molecules are able to cross the blood–brain barrier (BBB) and that the gut microbiome may therefore influence neurodevelopmental trajectories. One example is *S*-adenosyl methionine, a compound which is most abundant in the neonatal forebrain and that acts as an essential methyl donor for DNA and histone methylation, a fundamental process for brain development and function (Swann et al., 2020).

It was shown that neurogenesis is influenced by microbiota since adult GF mice exhibit increased neurogenesis in the dorsal hippocampus compared to conventional laboratory mice (Ogbonnaya et al., 2015). This phenotype could not be reversed by colonization of GF mice at weaning and indicated that microbial signals very early in life reduce the rate of neurogenesis in the hippocampus. Further, GF mice differ in dendrite morphology and show increased volume of the amygdala and the hippocampus (Luczynski et al., 2016). Moreover, decreased neurogenesis in the hippocampus of adult mice was induced after long-term antibiotic treatment leading to deficits in the novel object recognition task. Treatment with probiotics and voluntary exercise were sufficient to rescue these phenotypes (Möhle et al., 2016).

Myelination of the peripheral and central nervous system occurs rapidly during early life and is critical in regulating motor, sensory as well as cognitive functions. Neonatal antibiotics (Abx)-induced dysbiosis dysregulates host-microbe interactions and myelination in the brain, thereby leading to decreased hippocampal neurogenesis, increased myelination in the pre-frontal cortex (PFC) and causes behavioral impairments of adult mice. The administration of the SCFA butyrate restored behavior and myelination impairments, suggesting a critical role of the gut microbiota in mediating these effects (Keogh et al., 2021).

In the last decade, there was a great number of results from studies using GF mice pointing to a key role of the commensal gut microbiota in early brain development and adult neurogenesis. These results confirmed – amongst others – the impact of microbiota on microglia maturation mediated via SCFAs and the modulation of astrocyte activity via tryptophan and aryl hydrocarbon receptors. The microbiota also influences the activation of peripheral immune cells and the cytokine profile which affects – beside systemic and CNS inflammation – brain development (Quigley, 2017).

Recently it was shown, that the meninges, the membranes that surround the brain and spinal cord, contain IgA-secreting plasma cells that are educated in the gut. IgA traps microbes in the intestinal mucus to prevent a breach of the mucosal layer (Moor et al., 2017) and it was elucidated that they exert the same function at the dural venous sinuses, an important internal barrier interface. Seeding the meninges with antibody-producing cells recognizing gut commensals likely ensures a defense against the most common invaders originating from the gut, thereby preventing bacteremia in the CNS. Therefore, IgA cells emerging from the gut may act as an immunological firewall which is assembled during homeostasis to prevent spreading of pathogens into the meninges and the underlying CNS parenchyma during gut inflammation and breach of the intestinal barrier (Fitzpatrick et al., 2020).

In summary, current data clearly indicate that signals from the microbiota can regulate neurogenesis, apoptosis and synaptic pruning. Thus, the development of a healthy and functional brain depends on many pre- and post-natal events that integrate various environmental cues like molecular signals from the gut, largely originating from its microbiome that are communicated via the bidirectional gut-brain axis.

## The Impact of the Gut Microbiome on the Homeostasis and Aging of the Nervous System

In addition to a healthy neuronal development, there is growing evidence that the commensal gut microbiota also highly influences brain function and architecture under homeostatic conditions and during normal aging.

It was demonstrated that the BBB integrity in the frontal cortex, hippocampus and striatum is influenced by the commensal gut microbiota. Germ-free mice display an increased permeability of the BBB, this phenomenon is already evident during embryonal development due to reduced tight junctions by decreased expression of claudin 5 and occludin (Braniste et al., 2014). Recolonization of GF mice with complex commensal gut microbiota or the application of the SCFA butyrate restored the integrity of the BBB. Further, it was recently shown that the host microbiota controls microglia maturation and brain innate immune function. Adult GF mice show stunted microglia under homeostatic conditions compared to microglia from SPF mice (Erny et al., 2015). Microbial derived SCFAs are known to be able to cross the BBB (Huuskonen et al., 2004). The oral application of a mixture of the three SCFAs acetate, propionate and butyrate was sufficient to drive maturation of microglia, which suggests that they may affect microglia directly (Erny et al., 2015). Additionally, it was also shown that commensals have an effect on adult hippocampal neurogenesis leading to more immature neurons in GF mice but not in conventionally colonized mice (Marín-Burgin and Schinder, 2012; Ogbonnaya et al., 2015).

Normal aging is characterized by a progressive functional cognitive decline (Partridge, 2001) accompanied by significant changes in the gut microbiota of the elderly (Claesson et al., 2011). How aging and gut microbiota dysbiosis, which also occurs naturally while aging, reshapes brain immune cell plasticity and homeostasis is still not fully understood. Functional and phenotypical plasticity of brain immune cells contribute to brain tissue homeostasis as well as disease and is profoundly influenced by its microenvironment and systemic factors. Circulating metabolites and plasma cytokine composition are significantly altered by gut dysbiosis leading to dysregulation of the peripheral immune system (Arpaia et al., 2013; Bachem et al., 2019; Lehallier et al., 2019). CNS immunity and neuroinflammation is also indirectly altered by dysbiosis through gut microbiota-derived signaling molecules (Erny et al., 2015; Dinan and Cryan, 2017; Ma et al., 2019). There are emerging findings in animals and humans suggesting that the gut microbiota play a major role in gut-brain communication, ultimately shaping neurological aging trajectories by either helping to maintain nervous system function into late life or promoting pathology and positions the intestinal microbiota as an arbiter of age-related neurological decline (Madison and Kiecolt-Glaser, 2021). Gut microbiota dysbiosis can induce aberrant immune responses, which in turn disrupt the local and systemic homeostasis of the host and emerging evidence has highlighted the importance of gut microbiota in age-related diseases of the central nervous system such as AD and stroke. Several approaches like manipulating the microbiome via fecal microbiota transplantation (FMT), administration of prebiotics and probiotics as well as dietary interventions have been utilized to reduce age-related dysbiosis in experimental models and in clinical studies (Holmes et al., 2020).

Cellular indexing of transcriptomes and epitopes by sequencing (CITE-seq) was used to map brain immune cell plasticity in response to systemic perturbations of aging and gut dysbiosis to characterize the compositional and transcriptional plasticity of brain immunity. CITE-seq revealed transcriptional changes among inflammatory/patrolling Ly6C^+^ monocytes and CNS-associated innate lymphoid cells (ILCs). The discovery of such immune cell plasticity during aging and gut dysbiosis can help to learn more about the critical components determining brain immunity in aging and to ascertain the onset of age-related neurodegenerative diseases (Golomb et al., 2020).

A recent study investigated the effect of aged gut microbiota on cognitive decline by using fecal microbiota transplantation from aged to young rats (Li et al., 2020). Their results exhibited that FMT impaired the cognitive behavior in the young recipient rats leading to decreased regional homogeneity in the medial prefrontal cortex and hippocampus, reduced expression of brain-derived neurotrophic factor (BDNF), N-methyl-D-aspartate receptor NR1 subunit and synaptophysin, changed synaptic structures and decreased dendritic spines and increased expression of advanced glycation end products (AGEs) and receptor for AGEs (RAGE). Additionally, following FMT young rats showed increased levels of oxidative stress and pro-inflammatory cytokines, indicating that oxidative stress and inflammation may underlie gut-related cognitive decline in aging.

Taken together, these studies point toward a critical involvement of gut microbiota in maintaining neuronal homeostasis as well as in normal and pathological aging. A well-balanced equilibrium between gut microbiota and the host appears to be essential in maintaining neuronal health while significant disturbances of this relationship can lead to a variety of psychiatric and neurological diseases, which we will discuss throughout the following paragraphs.

## The Impact of the Gut Microbiome on Disorders of the Central Nervous System

**FIGURE 2:**
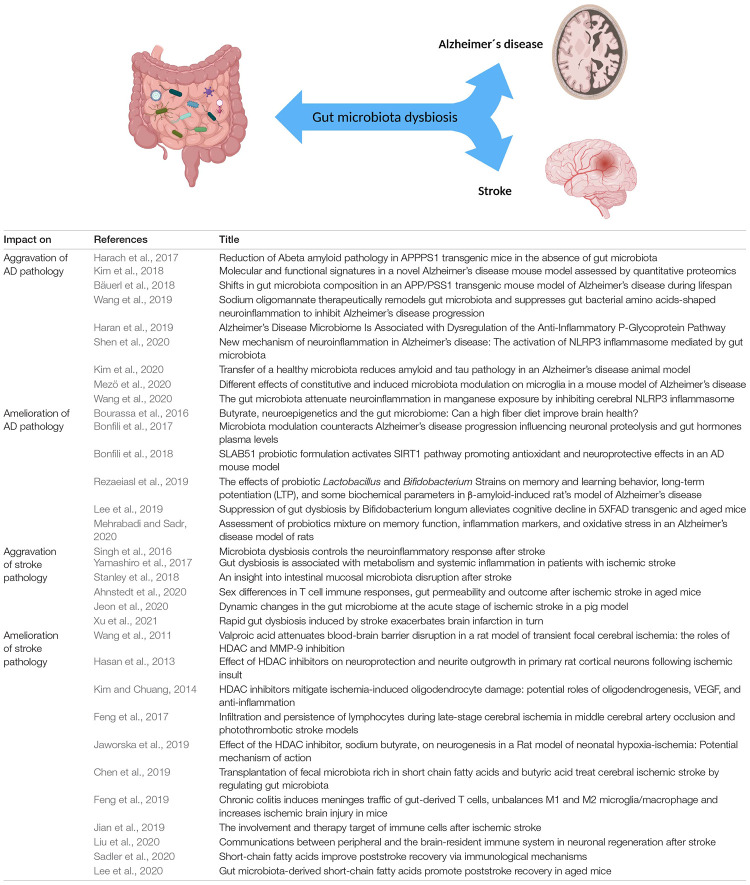
Reciprocal effects of the gut microbiota on Alzheimer’s disease and stroke. Numerous studies highlight that gut microbiota influences various CNS disorders such as Alzheimer’s disease and stroke in a negative and/or positive fashion. CNS disorders can also act on gut microbiota composition via the gut-brain axis. Table lists corresponding references, all studies are primarily based on rodent data with the exception of reference (Yamashiro et al., 2017; Haran et al., 2019; and Jeon et al., 2020).

After discussing the impact of the microbiome on the healthy development, the homeostasis as well as normal aging of the nervous system we will now focus on how microbiota influence the two most common neurological diseases that likely impose the greatest socioeconomic burden on humanity in the field of neuroscience: Alzheimer’s disease and stroke (Figure 2). In this context we will illustrate that our current mouse models were an excellent tool for proof-of-principal studies and therefore crucial in gaining fundamental insights into pivotal host-microbiota interactions. However, we will also discuss their weaknesses in the one-to-one translation of mouse model-based results into human clinical trials. Last but not least and based on the lessons learned throughout this review we propose a strategy that may increase the translational research value of current mouse models and that may help us to tap into the full therapeutic potential of translational microbiome research in the field of neuroscience.

### Microbiota and Alzheimer’s Disease

According to the World Health Organization AD is the most common form of dementia and by far the greatest factor causing disability and dependency among older people around the world (WHO, 2020). The clinical picture of AD has severe physical, psychological, social and economic effects, not only on people with AD, but also on their caregivers, families and the society as a whole; the socioeconomic consequences are dramatic and cannot be overstated. However, we still have no effective prevention and treatment strategy for AD (Zahs and Ashe, 2010; Cummings et al., 2014).

Alzheimer’s disease is a degenerative disorder caused by polymerization of amyloid β leading to the progressive loss of neurons. Interestingly, it has been recognized that amyloid β can act as an antimicrobial peptide via activation of toll-like receptor 2 (Welling et al., 2015; Kumar et al., 2016). However, it possesses harmful properties in a dysregulated state. Gut microbiota are a source of amyloid proteins sharing some structural similarities with amyloids from the CNS. The immune system could be primed by bacterial proteins in the gut enhancing immune response to endogenous productions of neuronal amyloid in the brain or they may act via molecular mimicry as prion proteins eliciting cross-seeding (Kowalski and Mulak, 2019).

Chronic overexposure to the biometal manganese (Mn) leads to manganism and is reported to cause neurodegenerative disorders (Guilarte and Gonzales, 2015). Its neurotoxic ability can potentially induce alterations to neuropathological features like amyloid β and Tau aggregation, which induces neuroinflammation in an NLRP3 inflammasome dependent manner at the heart of AD and PD. It was recently shown, that Mn exposure exaggerates host amyloid β and Tau production in the brain and causes hippocampal degeneration and necrosis. Fecal microbiome transplantation from normal control rats into rats exposed to Mn resulted in reduced amyloid β and Tau expression and downregulated NLRP3 and other neuroinflammatory factors. Hence, remodeling the gut microbiota in Mn exposer could be used as a therapeutic strategy to dampen neuroinflammation (Wang et al., 2020).

It was shown, that the widely used APP/PS1 double transgenic mice that are expressing a chimeric mouse/human amyloid precursor protein (APP) and a mutant human presenilin 1 (PS1) had a remarkable shift in the gut microbiota diversity compared to non-transgenic wild-type mice. Additionally, germ-free APP/PS1 transgenic mice exhibit a drastic decrease in the level of cerebral amyloid β pathology versus healthy control mice with gut microbiota (Harach et al., 2017). Similar results in the shift of microbiota composition were observed in the transgenic APP/PS1 mouse model by Bäuerl and displays higher amounts of the inflammatory related *Erysipelotrichaceae* family (Bäuerl et al., 2018). Further, germ-free APP/PS1 mice were found to show a reduction in amyloid β pathology in contrast to conventional mice (Radde et al., 2006). Additionally, the ADLP^APT^ mouse model expressing amyloid precursor protein, presenilin 1 and tau protein carrying human mutations, develops an AD-like pathology with amyloid and neurofibrillary tangles (Kim et al., 2018). They also show community level-alterations in the gut microbiota compared to wild-type mice. FMT from wild-type donor mice was able to alleviate the formation of amyloid β plaques and neurofibrillary tangles, glial reactivity and cognitive impairment in the recipient animals (Kim et al., 2020). In another common AD mouse model that expresses five familial AD mutations (5xFAD), it was demonstrated that the anti-inflammatory *Bifidobacterium longum*, which is able to suppress human gut microbiota LPS production and LPS-induced NF-κB activation in LPS-stimulated microglial BV-2 cells, from healthy human fecal microbiota could alleviate cognitive decline in 5xFAD transgenic and aged mice (Lee et al., 2019). In an effort to investigate the different effects of constitutive and induced microbiota modulation on microglia, 5xFAD mice bred under both GF and SPF conditions as well as mice with induced gut bacteria depletion by ABX were used (Mezö et al., 2020). The results revealed that constitutive or induced microbiota modulation differentially controls microglial amyloid β clearance mechanisms and prevents neurodegeneration and cognitive deficits. Actually, the constitutive absence of gut microbiota in GF 5xFAD mice enhanced the microglial uptake of amyloid β deposits in the hippocampus which led to a decreased amyloid β burden and associated neuronal loss as well as retained hippocampus-associated memory function.

Further, it was shown in a recent study that gut microbiota from AD patients can promote intestinal inflammatory response by activating intestinal NLRP3 inflammasome (Shen et al., 2020). Gut microbiota from AD patients were transplanted into APP/PS1 double transgenic mice leading to increased expression of NLRP3 in the intestinal tract and increased expression levels of inflammatory factors in peripheral blood. In addition, activation of microglia in the central hippocampus of these mice as well as increased expression of neuroinflammatory factors was observed. Transplantation of healthy human gut microbiota was then used to improve the composition of gut microbiota leading to down-regulation of the intestinal expression of NLRP3, improved cognitive ability of the mice, suppressed activation of microglia in the central hippocampus and down-regulated expression of neuroinflammatory factors. Further, the intestinal expression of NLRP3 was upregulated after transplantation of gut microbiota from AD patients into C57BL/6 mice. Therefore, improving the composition of intestinal bacteria in AD patients can attenuate neuroinflammation caused by NLRP3.

Wang et al. (2019) investigated the dynamic changes of gut microbiota associated with neuroinflammation in AD with respect of microbial derived amino acid metabolites. They used 5xFAD and APP/PS1 mouse models and observed that alteration of gut microbiota composition leads to peripheral accumulation of phenylalanine and isoleucine, which are able to stimulate the differentiation and proliferation of pro-inflammatory T helper 1 cells (Th1). Once the peripheral Th1 cells have infiltrated the brain, they are associated with M1 microglia activation, which contributes to AD-associated neuroinflammation. Additionally, they used a clinical phase 3 sodium oligomannate drug that was shown to improve cognition to target the gut microbiota. This drug could suppress gut dysbiosis together with the associated phenylalanine/isoleucine accumulation, restrained neuroinflammation and reverses cognition impairment.

There is growing evidence that probiotics and prebiotics beneficially modulate microbial and immune pathways to improve neurological disorders and brain functions (Suganya and Koo, 2020). AD rats treated with probiotics for 4 weeks exhibit significantly improved spatial learning and memory, long-term potentiation, paired-pulse facilitation ratios and lipid profiles (Rezaeiasl et al., 2019) along with decreased amyloid β plaques and reduced oxidative and inflammatory markers within 10 weeks (Mehrabadi and Sadr, 2020). Another probiotic formulation used in AD mice attenuated cognitive impairment, brain injuries, amyloid β aggregation and alternation of neuronal proteolysis (Bonfili et al., 2017) plus antioxidant and neuroprotective effects via activation of the SIRT1 pathway (Bonfili et al., 2018). Additionally, it has been reported that prebiotics may improve brain function and prevents neurological disorders like AD (Kinney et al., 2018).

The SCFA butyrate is synthesized in the colon by the commensal gut microbiota via fermentation of otherwise non-digestible fiber (Pryde et al., 2002) and can improve brain health. Butyrate uses diverse modes of action to execute its beneficial effect on various brain disorders: (i) as energy metabolite to produce ATP playing a role in metabolism and mitochondria activity (e.g., in AD, PD, stroke, mitochondrial encephalopathy, and adrenoleukodystrophy), and (ii) its epigenetic ability to serve as a histone deacetylase inhibitor (e.g., AD, PD, and stroke). This wide array of biological functions makes butyrate to a very attractive therapeutic molecule to prevent neurodegeneration and to promote regeneration (Bourassa et al., 2016). Interestingly, elderly patients with AD carry a lower abundance of butyrate producing bacteria and instead a higher proportion of taxa that are associated with neurological disorders and taxa that are known to cause proinflammatory states. Furthermore, it was demonstrated that stool samples from elderly AD patients induced lower production of anti-inflammatory p-glycoprotein *in vitro* than samples from elders without AD or with other dementia types (Haran et al., 2019).

Around 50 million people worldwide suffer from dementia and there are almost 10 million new cases reported every year (WHO, 2020). Although AD is with 60–70% by far the most common cause of progressive dementia, there is still neither a prophylactic nor an effective therapeutic strategy against this devastating disease (Scheltens et al., 2016). The WHO estimates the current proportion of dementia cases in the population over the age of 60 years at approximately 5–8%. Importantly, the total number of dementia patients - according to current estimates - is expected to reach 82 million in 2030 and up to 152 million in 2050 (WHO, 2020), imposing a significant global health and socioeconomic burden on humanity that will, if it progresses uncontrolled, overwhelm our healthcare systems. This dramatic situation demands – from an economical, ethical and social perspective – the highest possible prioritization of research into AD and the fastest possible development of new and effective treatments. Microbiome research may have the inherent potential of providing answers, at the very least to some of these challenges. Most basic research on AD has predominantly used wildtype and genetically modified mice (GF, antibiotic-treated, gnotobiotic, and SPF) to recapitulate the genetic and pathological elements of human AD, thereby deriving mechanistic and therapeutic knowledge that is hoped to translate to the human condition. The translational goal of basic research for medical diseases in all fields is to provide the knowledge required to predict, prevent, identify, treat, and hopefully cure these diseases in human patients. As such, the success of this approach for AD is rather poor. For instance, as reviewed by Zahs and Ashe, over 200 different interventions have been reported to be effective in the APP mouse model of AD, but none has proven effective in human trials (Zahs and Ashe, 2010). In fact, the rate of AD drugs entering human trials and fail was 96.4% from 2002 to 2012 (Cummings et al., 2014) leading to the emerging challenge of producing animal based results that better translate to human outcomes.

As already described by scientists in 1989, Alzheimer’s patients exhibit plaque deposits not only in the brain, but also in the intestine (Joachim et al., 1989), indicating a linkage of both organs in the context of AD. With age, the integrity of the BBB and the gastrointestinal epithelium decline (Tran and Greenwood-Van Meerveld, 2013) as well as the composition of the gut microbiome (Woodmansey, 2007) changes with age, and it is known that age is the greatest risk factor for AD. In addition, many genes associated with AD are expressed in the gut and several microbial factors have been linked to AD pathogenesis (Brandscheid et al., 2017). This indicates that we need mouse models with gut microbiota and immune systems that will allow scientists to better translate the findings of gut microbiota-AD interaction into the human system.

### Microbiota and Stroke

According to the WHO, the cerebrovascular accident also known as stroke is the second leading cause of death and the third leading cause of disability worldwide (WHO, 2012). There are over 13.7 million new cases of strokes reported each year (GBD 2016 Stroke Collaborators, 2019) and globally, one in four people over the age of 25 will have a stroke in their lifetime (Feigin et al., 2018). Since the gut microbiome is known to significantly influence vascular diseases, its impact on the cerebrovascular disease stroke appears obvious and logical as reviewed elsewhere (Tang et al., 2017; Durgan et al., 2019; Battaglini et al., 2020; Verhaar et al., 2020). Moreover, this connection comes with the inherent potential of developing microbiota-related therapeutics.

In the context of stroke, the delicate interplay of the bidirectional communication between the gut microbiota and the CNS along the gut-brain-axis is also widely discussed leading to the question whether gut dysbiosis is the cause or the consequence of stroke. Post stroke, the commensal microbiota change in favor of opportunistic pathogens, which may be due to the systemic release of cytokines and chemokines produced in the brain, changed gut motility and permeability as well as mucus production leading to dysbiosis. Dysbiosis subsequently worsens the outcome of stroke. However, gut dysbiosis has also been associated to reinforce risk factors for acute ischemic stroke like hypertension, diabetes, obesity, vascular dysfunction or the changed microbiota during aging (Battaglini et al., 2020).

Several animal-based studies have demonstrated substantial differences in microbiota composition even at the phylum level between poststroke models and controls in all sections of the gastrointestinal tract like increased abundance of *Akkermansia muciniphila* and clostridial species (Stanley et al., 2018). Changes of gut microbiota were already visible within 3 days after stroke (Singh et al., 2016) and included a reduced diversity of microbiota species, intestinal bacterial overgrowth with a preferential expansion of the Bacteroidetes phylum, and more specific stroke-induced changes on the bacterial genus and at the species level (Yamashiro et al., 2017).

Since the elderly experience profound systemic responses after stroke resulting in more severe long-term disability and higher mortality, a recent study investigated if restoring youthful gut microbiota after stroke benefits recovery in aged subjects (Lee et al., 2020). Gut microbiota from young mice were gavaged into aged mice 3 days after induction of ischemic stroke resulting in less behavioral impairment as well as reduced brain and gut inflammation. Microbial sequencing and metabolomics analysis revealed that young fecal transplants contained much higher SCFA levels and four related bacterial strains were used for a more defined transplantation. The four selected SCFA-producers were able to attenuate post-stroke neurological deficits and inflammation in aged stroke mice. In addition, they were able to boost concentrations of SCFA in the gut, the plasma as well as the brain. These results suggest that the poor stroke recovery in aged mice can be reversed via post-stroke bacteriotherapy of youthful gut microbiota.

Observation of a clinical cohort of patients with ischemic stroke revealed a fast induction of intestinal ischemia and an excessive production of nitrate resulting in gut dysbiosis with expansion of *Enterobacteriaceae* (Xu et al., 2021). Overgrowth of *Enterobacteriaceae* magnifies brain infarction by enhancing systemic inflammation and is a potential risk biomarker for a poor primary outcome of patients. A mouse model with middle cerebral artery occlusion also showed fast gut dysbiosis based on stroke-induced intestinal ischemia and reperfusion leading to nitrate respiration causing enrichment of *Enterobacteriaceae*. The increase of *Enterobacteriaceae* accelerates systemic inflammation through the LPS-TLR4 pathway and aggravated brain infarction. The overgrowth of *Enterobacteriaceae* could be suppressed by blocking nitrate generation or nitrate respiration which reduced the systemic inflammation and further ameliorates brain infarction.

Interestingly, stroke displays some well-known sex differences. Recent data adjusted for age and pre-stroke functional status revealed that while women account for more stroke deaths, men display a higher overall mortality. A current study investigated in aged mice the sex differences in immune response, gut permeability and microbial diversity after stroke induction. In males, stroke induced greater gut permeability and non-reversible alterations in microbiota diversity along with greater neurological deficits and impaired sensorimotor function as well as greater cognitive decline (Ahnstedt et al., 2020).

A recent study utilizing a middle cerebral artery (MCA) occlusion ischemic stroke pig model evaluated the changes in gut microbiota composition and diversity past stroke (Jeon et al., 2020). After ischemic stroke induction, blood and fecal samples were taken at different time points and revealed ameliorated systemic inflammation with elevated plasma levels of TNF-α and IL-6 12 h after the stroke. The microbial diversity was already reduced at day 1 post stroke and almost normalized by day 5 indicating dynamic changes and plasticity of the gut microbiome in an acute period of stroke.

As shown for AD, intestinal microbiota can also benefit stroke outcome. Gut microbiota can promote neuronal regeneration via immune-related signaling and metabolites that can directly alter the phenotypes of resident immune cells. Circulating immune cells that have been shaped by immune components including the gut microbiota and their metabolites can infiltrate the brain and influence neuronal regeneration directly or through modulation of the properties of brain-resident immune cells in an ischemic brain (Liu et al., 2020). Thereby, gut microbiota and associated metabolites could be a component of a repair system after stroke that can strengthen repair-promoting immune responses or weaken neurotoxic immune responses to promote neuroplasticity (Feng et al., 2017; Jian et al., 2019).

Since it was shown that lymphocytes migrate from the gut to the brain after stroke, the altered gut microbial composition after stroke may shape the local immune environment in the brain in favor of neurogenesis and axon growth. It was observed that gut-derived CD4^+^ T cells migrate after an ischemic injury to the meninges and control the balance between M1 and M2 microglia/macrophage (Feng et al., 2019). SCFAs can activate and recruit T cells to the brain, which then regulate the function of brain-resident microglia to promote synaptic plasticity after stroke in male mice (Sadler et al., 2020). Additionally, SCFAs can stimulate phenotype transition of microglia and exert neurogenesis and neurite outgrowth permissive effects under ischemic conditions (Hasan et al., 2013; Jaworska et al., 2019). Butyric acid supplementation or SCFA-enriched fecal microbiota transplantation have both been shown to promote neurological recovery in adult neurogenesis after stroke (Chen et al., 2019). Further, sodium butyrate is able to reduce the infarct size in ischemic stroke models along with limited brain damage and improved behavioral outcomes (Kim et al., 2007; Langley et al., 2008; Wang et al., 2011; Kim and Chuang, 2014).

Taken together, gut microbiota is also a hot topic in the field of stroke research and was already critically discussed in the early days of stroke-microbiota research. Back then, researchers raised awareness for the fact that the gut contains not just bacteria but also viruses and bacteriophages, that most research is performed in GF mice and that we have to consider that they may deviate from normal physiology and have underdeveloped immune structures that might deceive results. Indeed, stroke research is highly struggling with a poor clinical translation rate and discussed in depth elsewhere (Pound and Rebecca, 2020). The vast majority of interventions for stroke successfully tested in preclinical animal studies have turned out to either have no efficacy (Horn and Limburg, 2001; Shuaib et al., 2007) or to be harmful to humans (Saxena et al., 1999; Bath et al., 2000; Davis et al., 2000; Enlimomab Acute Stroke Trial Investigators, 2001) and increased the risk of death in clinical trials. From more than 1,000 candidate neuroprotective drugs that where tested in animals, not a single one was found to benefit humans with stroke (O’Collins et al., 2006) and animal studies were just used to establish dosing but did not play a direct role in clinical translation (Pound and Rebecca, 2020). Additionally, just 2 out of approximately 500 compounds that have been reported to effectively reducing the effects of acute ischemic stroke in animal models have proven to be effective in humans. Besides drug development, behavioral studies continue the translational disappointments: While voluntary physical activity improves long-term stroke outcome in mice (Gertz et al., 2006), a higher rate of serious adverse events was observed in the aerobic group of subacute stroke patients compared with a relaxation group that received relaxation sessions (Nave et al., 2019). Thus, also the field of stroke urgently needs animal models that better recapitulate the human system for a more successful transfer from interventions from basic research and preclinical animal studies to human clinical trials.

## Concluding Remarks

Finally, we want to propose future strategies that may help to tap into the full therapeutic potential of translational microbiome research in the field of neuroscience.

Particularly, the mammalian gut microbiome produces a recently unappreciated, but remarkably diverse set of biologically active products such as small molecule metabolites and their precursors. These compounds do not only exert local effects within the gut, they are also capable of breaching the intestinal barrier subsequently affecting various gut-distal sites such as the nervous system as described throughout this review. It is likely that these molecules play a dominant role in orchestrating the communication along the gut-brain axis (Diaz Heijtz et al., 2011; Collins et al., 2012; Wu et al., 2012; Forsythe et al., 2014; Sampson and Mazmanian, 2015; Yano et al., 2015; Kipnis, 2018). Hence, translational microbiome research, especially in the field of neuroscience, should aim to identify these compounds and to understand their biological function in health and disease. This approach may open up a promising window of opportunity and may be a pathbreaking way to discover novel drugs for the treatment of a broad variety of human diseases (Brown and Hazen, 2017).

In this context, it is pivotal to emphasize that the gut microbiota is an extremely complex and diverse ecosystem comprising microorganisms of all kingdoms – not just bacteria – that communicate with their host and with each other through biologically active compounds in a multifactorial and non-linear way (Norman et al., 2014; Pfeiffer and Virgin, 2016; Stappenbeck and Virgin, 2016). This was illustrated by several recent studies highlighting the impact of non-bacterial organisms of the microbiome on host physiology such as protists (Chudnovskiy et al., 2016), parasites (Howitt et al., 2016), fungi (Ackerman and Underhill, 2017), and viruses (Lim et al., 2016). Consequently, experimental models of translational microbiome research that are aiming to decipher mechanisms should benefit from a more complete description of the microbiome. Nevertheless, most studies still keep their focus exclusively on the bacterial microbiome, merely one component of the entire microbiome, which makes it not only difficult to decipher complex microbiota-related mechanisms, but also inherently restricts our chances of identifying novel, biologically active microbial compounds. Thus, a full description of the microbiome and a more comprehensive view of mammalian organisms as holobionts might be a key for the development of novel microbiota-based therapeutics.

Germ-free, antibiotic-treated, gnotobiotic, and SPF mouse models were essential to unravel basic principles as regards to the importance of gut microbiota in development, maturation, aging, homeostasis as well as function of the brain in health and disease. These lessons learned were fundamental in illuminating the therapeutic potential lying within microbiome research such as the use of microbial bio-active metabolites in the treatment of neurological diseases. However, the overwhelming amount of rodent-based data could not be directly translated from bench to bedside practice (Fisher et al., 1996; Mestas and Hughes, 2004; von Herrath and Nepom, 2005; Suntharalingam et al., 2006; Payne and Crooks, 2007; Seok et al., 2013; Shay et al., 2013; Garner, 2014; Hay et al., 2014; Mak et al., 2014; Wong et al., 2019; Pound and Rebecca, 2020) as we have demonstrated for AD (Zahs and Ashe, 2010; Cummings et al., 2014) and stroke (Saxena et al., 1999; Bath et al., 2000; Davis et al., 2000; Enlimomab Acute Stroke Trial Investigators, 2001; Horn and Limburg, 2001; Gertz et al., 2006; O’Collins et al., 2006; Shuaib et al., 2007; Nave et al., 2019; Pound and Rebecca, 2020). This raises the question of how the scientific community could optimize translational research models to better reflect human physiology in health and diseases. Given the vast impact of the microbiome on the immune and nervous system, it appears likely that the field of microbiome research might help to address these issues. In this context, recent paradigm-shifting work illustrated that conventional laboratory mice are too far removed from natural environmental conditions to faithfully mirror the physiology of free-living mammals like humans. These mice lack the diverse microbial exposure that humans experience from birth and throughout their whole life, which is integral for immune maturation as well as immune experience and consequently the orchestration of immune responses. When working with conventional laboratory mice, this ultimately leads to false assumptions of how the human immune system works as reviewed elsewhere (Masopust et al., 2017; Graham, 2021).

In an effort to optimize mouse models of translational research, several approaches such as cohousing of conventional laboratory mice with pet store mice (Beura et al., 2016), sequential infections (Reese et al., 2016), rewilding in semi-natural habitats (Leung et al., 2018), engraftment of wild mouse gut microbiota into pregnant germ-free mice (Rosshart et al., 2017) and the transfers of conventional laboratory mouse embryos into wild mouse surrogate mothers (Rosshart et al., 2019) – the so-called wildling model – were proposed. Particularly wildlings accurately phenocopied the human outcome and could have prevented catastrophically failed clinical trials (Rosshart et al., 2019), where conventional laboratory mice as well as rat and non-human primate models had failed to predict the human response to harmful drug treatments (Fisher et al., 1996; Suntharalingam et al., 2006). Moreover, when compared to conventional mice, such animals were protected in models of various infectious diseases as well as cancer (Beura et al., 2016; Rosshart et al., 2017), which may allow for the identification of microbiota-mediated protective mechanisms that cannot be discovered in conventional laboratory mice. The idea of naturalizing mouse models for immunology to improve their translational potential was just recently discussed (Masopust et al., 2017; Graham, 2021).

Even though and likely due to the very recent appearance of these new mouse models, there is only limited direct evidence (Cope et al., 2019) that the use of such models will also improve the translational research value of mouse models in the field of neurosciences. Nevertheless, since we know that the microbiome and the immune system play an important role in mammalian physiology and particularly in health and disease of the CNS, it seems likely that the described models are a promising tool to also enhance the translational research value of mouse models in the context of neuroscience. Hence, adding these novel models to our experimental toolbox in neuroscience may facilitate the discovery of disease mechanisms and treatments that cannot be found in GF, antibiotic-treated, gnotobiotic and conventional SPF mice, increase the safety and success rate of bench-to-bedside efforts, reduce costs and ultimately accelerate the development of new disease treatments.

In conclusion, based on the profound knowledge scientists gained as regards to the impact of microbiota on the nervous system in health and diseases, we believe in the importance and therapeutic potential of microbiome research conducted in the context of neuroscience. However, to access the full therapeutic potential of microbiome research and subsequent drug discovery and development, we propose to (i) foster the holistic view on mammals as holobionts and to study all members of the microbiome as well as their biologically active products and metabolites and (ii) to take advantage of newly developed translational research models that more closely resemble the human meta-organism. This approach may help to unleash the full potential of microbiome research regarding the discovery of novel microbiota-related therapeutics of numerous neurological disorders of global relevance.

## Author Contributions

MS wrote the manuscript. MS and SR designed the figures. SR supervised and edited the manuscript. Both authors listed and approved it for publication.

## Conflict of Interest

SR discloses that Taconic Biosciences licensed WildR mice with natural gut microbiota from NIDDK. The remaining author declares that the research was conducted in the absence of any commercial or financial relationships that could be construed as a potential conflict of interest.

## Abbreviations

AD: Alzheimer’s disease
AGEs: advanced glycation end products
APP: amyloid precursor protein
ASD: autism spectrum disorder
BBB: blood–brain barrier
BDNF: brain-derived neurotrophic factor
CITE-seq: cellular indexing of transcriptomes and epitopes by sequencing
CNS: central nervous system
CPT1: carnitine palmitoyltransferase 1
FMT: fecal microbiota transplantation
GF: germ-free
ILCs: innate lymphoid cells
MAMPs: microbial-associated molecular patterns
MCA: middle cerebral artery
Mn: manganese
MS: multiple sclerosis
PD: Parkinson’s disease
PFC: pre-frontal cortex
PS1: presenilin 1
RAGE: receptor for AGEs
SCFAs: short-chain fatty acids
SPF: specific-pathogen-free
Th1: T helper 1
TMAO: trimethylamine N-oxide.
